# Prediction of 7-year psychopathology from mother-infant joint attention behaviours: a nested case–control study

**DOI:** 10.1186/1471-2431-13-147

**Published:** 2013-09-24

**Authors:** Clare S Allely, Paul CD Johnson, Helen Marwick, Emma Lidstone, Eva Kočovská, Christine Puckering, Alex McConnachie, Jean Golding, Christopher Gillberg, Philip Wilson

**Affiliations:** 1 Institute of Health and Wellbeing, University of Glasgow, RHSC Yorkhill, Glasgow G3 8SJ, Scotland; 2 Robertson Centre for Biostatistics, Boyd Orr Building, University of Glasgow, Glasgow G12 8QQ, Scotland; 3 National Centre for Autism Studies at the University of Strathclyde, Glasgow, Scotland; 4 Centre for Child and Adolescent Health, School of Social and Community Medicine, University of Bristol, Bristol, UK; 5 Centre for Rural Health, University of Aberdeen, The Centre for Health Science, Old Perth Road, Inverness IV2 3JH, Scotland

**Keywords:** Avon longitudinal study of parents and children (ALSPAC), Autism, Attention deficit hyperactivity disorder (ADHD), Disruptive behaviour disorders, Joint attention behaviours

## Abstract

**Background:**

To investigate whether later diagnosis of psychiatric disorder can be predicted from analysis of mother-infant joint attention (JA) behaviours in social-communicative interaction at 12 months.

**Method:**

Using data from a large contemporary birth cohort, we examined 159 videos of a mother-infant interaction for joint attention behaviour when children were aged one year, sampled from within the Avon Longitudinal Study of Parents and Children (ALSPAC) cohort. Fifty-three of the videos involved infants who were later considered to have a psychiatric disorder at seven years and 106 were same aged controls. Psychopathologies included in the case group were disruptive behaviour disorders, oppositional-conduct disorder, attention-deficit/hyperactivity disorder, pervasive development disorder, anxiety and depressive disorders. Psychiatric diagnoses were obtained using the Development and Wellbeing Assessment when the children were seven years old.

**Results:**

None of the three JA behaviours (shared look rate, shared attention rate and shared attention intensity) showed a significant association with the primary outcome of case–control status. Only shared look rate predicted any of the exploratory sub-diagnosis outcomes and was found to be positively associated with later oppositional-conduct disorders (OR [95% CI]: 1.5 [1.0, 2.3]; p = 0.041).

**Conclusions:**

JA behaviours did not, in general, predict later psychopathology. However, shared look was positively associated with later oppositional-conduct disorders. This suggests that some features of JA may be early markers of later psychopathology. Further investigation will be required to determine whether any JA behaviours can be used to screen for families in need of intervention.

## Background

Joint attention (JA) is important in early interaction as it is a fundamental aspect of interpersonal connection in typical social communicative development, underpinning referential understanding, shared concepts and perspective taking abilities (the ability to relate to others) as well as contributing to concordant inter-subjectivity [1-3]. During typical infant-caregiver interactions, there is the ability to engage in order to share attention to objects or events of mutual interest and JA provides a context in which mutual regulation of affect and of problem solving, for the negotiation of communicative intentions and for the sharing of cultural meaning, can take place [4,5]. Active social behaviour increases dramatically around 1–2 months of age as infants begin to engage in direct face-to-face interactions with adults [6-8]. Infant capacity for JA behaviours typically emerges between 6 and 12 months and involves the triadic interaction (‘jointness’) [9] of attention between the infant, another person (typically an adult) and a third object such as a toy [10-12]. JA is a term which characterises a wide range of behaviours including gaze and point following, showing and pointing. JA behaviours serve two different functions: imperative triadic exchanges serve an instrumental or requesting function, and declarative triadic exchanges which enables shared awareness or experience of an object or event [13,14].

The majority of research investigating JA surrounds its involvement in both cognitive and language development [15,16] while relatively little research has focused on the relationship of JA with social-emotional factors [17]. JA skill development has been found to be involved in early adaptive social–emotional behavioural development [18-20]. Since children who exhibit language development difficulties are at increased risk of behavioural and emotional disorders [21-23], infant JA skills may be associated with both language development and social behavioural development. A number of JA behaviours in infancy are signs of processes associated with self-monitoring, emotional reactivity and prosocial affiliative tendencies [24,25] which are behavioural dimensions found to be associated with the emergence of social competence in young children [26].

Recently, global characteristics of parent–infant interaction in 6–10 month-old at-risk and low-risk infants were examined using six minute videos of unstructured mother-infant play. At-risk infants were found to be less lively, and their parents exhibited both higher directiveness and lower sensitive responding [27]. Marwick et al. [28] recently demonstrated, using a holistic analysis of interpersonal behaviours within early social interaction, that lower levels of adult activity and adult speech predict later psychiatric diagnosis in the child at seven years of age. Analysis of the infants’ interactive behaviours revealed no predictors of later psychopathology.

### Present study

Particular patterns of parent-infant interactions can aid prediction of later development of childhood psychopathology including attention deficit hyperactivity disorder (ADHD) and autism [2]. JA abilities are crucially involved in the development of autism with impairments in JA amongst the earliest signs of the disorder [29-36]. JA difficulties in relation to the external environment is argued to be an indicator or precursor for other adverse consequences in childhood: disruptive behaviour [17,37], disturbances in language development [36] and disturbances in learning and social cognition [38-40]. Disturbances in the parent–child relationship in early childhood are known risk factors for later psychological maladjustment [41]. There is a need for examination of JA behaviours in adult-infant interaction to establish if these are predictive of later diagnosis of social communicative disorder to enable early identification and support. Identification of infant predictors of later childhood psychopathology is important for informing appropriate and timely intervention.

Based on videoed caregiver-infant interactions from a large population-based birth cohort, we examined whether analysis of mother-infant joint attention behaviours in social-communicative interaction at 12 months are predictive of later diagnosis of psychopathology in the child at seven years of age. To our knowledge, this is the first study which has examined whether mother-infant joint attention behaviours during an interaction when the infant is 12 months is predictive of later diagnosis of a wide range of psychopathologies, not simply autism, using a nested case–control study.

## Method

### Participants

Participants were selected from the Avon Longitudinal Study of Parents and Children (ALSPAC) which is an ongoing population-based study investigating a wide range of environmental and other influences on the health and development of children. Pregnant women resident in the former Avon Health Authority in south-west England, having an estimated date of delivery between 1 April 1991 and 31 December 1992 were invited to take part, producing a ‘core’ cohort of 13,988 singletons/twins alive at 12 months of age [42]. Please note that the study website contains details of all the data that is available through a fully searchable data dictionary (http://www.bris.ac.uk/alspac/researchers/data-access/data-dictionary/).

Ethical approval was obtained from the ALSPAC Law and Ethics Committee and the Local Research Ethics Committees for the present study. All adult participants gave their informed consent prior to their inclusion in the study. For the current study a sample was drawn from a sub sample of the core ALSPAC cohort who were invited to attend Children in Focus clinics after birth. 1240 participating families (usually mother/infant dyads) attended the clinic which took place when the children were 12 months old. One of the sessions at the clinic involved the Thorpe Interaction Measure (TIM) [43]. In this session, the mother was asked to share a picture book with her child and engage him/her in this activity as they would at home. All interactions took place in the same ‘living room’ style environment in the clinic and were recorded on videotape. If the child became distressed or was indicating that he/she had had enough, video recording was terminated. The static camera recording the caregiver-infant interaction was placed in the upper corner of the room. As a result of this, the caregivers’ and infants’ faces were occasionally not visible, making some judgments difficult. The mean duration of these caregiver-infant interactions was 4.3 (SD 2.6) minutes with a range from 1.5 to 17.2 minutes.

**Figure 1:**
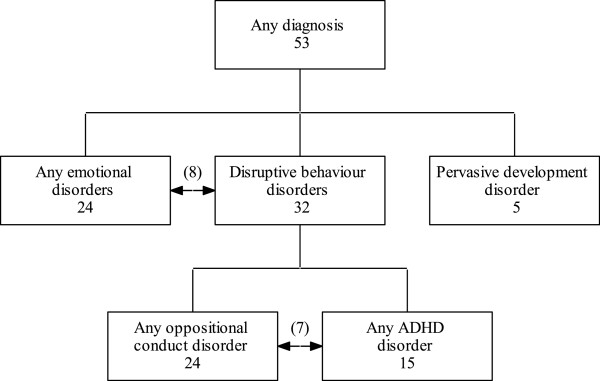
Flow chart of the diagnostic outcomes of the 53 cases using the Development and Wellbeing Assessment (DAWBA) at age seven.

We report a case–control study nested within a prospective cohort study. The sample comprised 159 mother-infant pairs (53 cases and 106 controls), reduced from 180 (60 cases and 120 controls) due to the exclusion of 19 videos where we judged that the lead carer was not the mother (the lead carer was the father in 17 and the grandmother in two of the videos), and two videos that could not be coded due to poor quality. The 53 cases were infants who were later assessed to have autism, conduct disorder, ADHD, anxiety or depression using the Development and Wellbeing Assessment (DAWBA) [44] which was administered to all children remaining in the cohort at 91 months of age (See Figure 1). The 106 controls used in this study were drawn from the original 120 randomly selected sex-matched controls. The DAWBA is a structured diagnostic assessment which relies on parental report as well as teacher reports, but final diagnoses are assigned by a child psychiatrist (see Table 1). Diagnostic categories were as follows: disruptive behaviour disorders (ADHD and/or any oppositional/conduct disorder), oppositional-conduct disorders (either conduct disorder, oppositional-defiant disorder or disruptive behaviour disorder-not otherwise specified (DBD-NOS)), any ADHD (either combined, inattentive or hyperactive-impulsive type), pervasive developmental disorder (autism), or any emotional disorder (anxiety, depression or phobias). Some infants later went on to develop more than one of the psychopathologies investigated here - eight were comorbid with disruptive behaviour disorders and emotional disorders. Of these, five had diagnoses of emotional, oppositional-conduct and ADHD disorders and three were comorbid with emotional disorders and oppositional-conduct disorders. A further two had diagnoses of both ADHD and oppositional – conduct disorders.

**Table 1:** Number of cases within each diagnostic group, overall and by gender

		**All**	**Female**	**Male**
		**(n = 159)**	**(n = 48)**	**(n = 111)**
Control		106	33	73
Case	All	53	15	38
	Disruptive behaviour disorders (any ADHD + any oppositional-conduct disorder)	32	6	26
	Any ADHD disorder	15	1	14
	Any oppositional-conduct disorder	24	5	19
	Pervasive development disorder	5	0	5
	Any emotional disorder	24	11	13

### Procedure

The videos of mother-infant interactions were examined using Noldus Observer software, by independent observers, blind to the later outcome of the children. Twenty three different elements (including, Joint Attention; Infant Gaze at Book; Infant Following Adult Focus; Infant Proto-declarative Pointing; Infant Pointing; Infant Smile; Infant Non-Response to Name and Infant Imitation) were examined. However, for the present study we examined only one of these: joint attention since previous literature has found joint attention behaviours to be significant in predicting later psychopathologies such as autism and other behaviours proved too infrequent to be useful in the analysis or not reliably coded in observations. We planned to assess the predictive utility of the tendency of infants to respond to their names, but did not have adequate power as only 34 (12 cases and 22 controls) of the 159 infants were called by their names and had the opportunity to respond. A more structured interaction setting, where mothers are directed to call the infant by name, might be required to investigate this question. Definitions of each of the elements can be found in Additional file 1 which can be found in the Supplemental Materials (online only).

Joint attention is the state of shared awareness of self and other’s attention, purposes, feelings and meanings in relation to a shared focus (to an object, action, event, experience or person). In this present study this was assessed through sequences of observable behaviours. Joint attention can be judged by observing a sequence of events in which:

1. The child and parent look at each other (initiation phase);

2. Followed by both looking at or acting on, or acting in relation to, a joint object or event (shared attention/action/communicative act phase);

3. Followed by the child and parent looking back at each other (confirm/check joint response phase).

For behaviours to be coded as joint attention they need to be consecutive. So joint attention would be coded if 1, then 2, then 3 was observed Alternatively, if 1 and 2 or 2 then 3 were observed then this would also be coded as joint attention.

### Statistical methods

Four measures of joint attention were calculated: number of shared looks per minute (shared look rate); percentage time spent in shared looks (shared look intensity); number of periods of shared attention per minute (shared attention rate); and percentage time spent in shared attention (shared attention intensity). Rates were calculated as (number of events ÷ duration of video in minutes) and intensities as (100 × shared duration ÷ total video duration). In order to assess the inter-rater reliability of the joint attention measures, a randomly selected subset of six videos were rated twice by two raters. As the use of weighted kappa to assess reliability was not justified due to non-normality, reliability for each measure was assessed by estimating Kendall’s τ [45] between the two raters. Strictly, Kendall’s τ gauges concordance among the ranks, not the measures themselves, but we justify its use on the grounds that non-parametric measures of true reliability (i.e. concordance) are not available. Measures with τ > 0.6 were considered reliable.

We expected positive correlations among the four measures. To avoid analysing highly correlated and therefore redundant variables, we assessed inter-measure correlation using Spearman’s ρ, judging variables with |ρ| > 0.5 to be at least moderately correlated.

### Estimation of odds ratios

We used logistic regression to investigate the degree to which joint attention scores from videos recorded at 12 months predicted diagnosis of psychopathologies at seven years. Odds ratios (ORs), 95% confidence intervals and p-values were estimated using Firth’s penalised likelihood logistic regression [46], implemented in the logistf package for R [47]. The primary analysis was a test of association between each joint attention measure and case–control status. Sub-diagnoses were tested as exploratory outcomes: disruptive behaviour disorders (any ADHD and/or any oppositional-conduct disorder); any ADHD disorder; any oppositional-conduct disorder; pervasive development disorder; any emotional disorder (anxiety and/or depression).

### Adjustment for potential confounders

All models were adjusted for sex and other potential confounders of the joint attention-psychopathology relationship. The following variables were considered as potential confounders: birthweight; weight, length and ponderal index at 12 months; the mother’s age at delivery; maternal depression measured using the Edinburgh Postnatal Depression Scale (EPDS) [48] at 32–40 weeks gestation and eight months postnatally; maternal and paternal social class of the infant (defined by non-manual or manual occupation each based on three levels I|II|III (non-manual)|III (manual)|IV|V); and the log of the duration of the interaction session. An association between the duration of the interaction and the outcome and/or the joint attention measures was plausible because the protocol allowed/obliged the sessions to be terminated early if the child became bored or distressed. There is a possible link in that if the mother or child is failing to make an interactive link the child may have become bored quicker. A variable was considered to be a confounder if it was associated (*p* < 0.1) with a joint attention measure in a generalized linear model (GLM) adjusted for case–control status and sex. Event rates were fitted as counts in a negative binomial GLM with an offset to adjust for video duration. Intensities were analyzed using ordinary least squares linear regression. For the confounder analysis only, intensities were transformed by squaring to give normally distributed residuals.

### Statistical power

We estimated that a test of the null hypothesis of no association (odds ratio = 1) between joint attention and case–control status would have 80% power to detect odds ratios of 1.7 per standard deviation difference in shared look rate, 2.0 with shared look intensity, 1.6 with shared attention rate and 1.8 with shared attention intensity, assuming a sample size of 53 cases and 106 controls. Odds ratios of 1.6 to 2.0 imply that an infant with a moderately high score of 1 SD above average would be at 60-100% higher risk of diagnosis of psychopathologies at seven years than an infant with an average score. Thus, this study is powered to detect strong associations between joint attention behaviours and psychopathology which have previously been found in young children [49].

## Results

Correlation between the raters was high for three of the four measures, consistent with high inter-rater reliability. Estimates of Kendall’s τ for shared look rate, shared attention rate and shared attention intensity were 0.83, 1.00 and 0.87, respectively, so these three measures were taken forward for further analysis. Kendall’s τ for “shared look (% time)” was low at 0.41, suggesting relatively poor reliability, and this measure was not analyzed further. There were no strong correlations between the three reliable measures (all |Spearman’s ρ| < 0.5), suggesting low redundancy. Summary statistics for the three reliable joint attention measures are presented in Table 2.

**Table 2:** Summary statistics for the three reliable joint attention measures in controls and cases

		**Control (n = 106)**	**Case (n = 53)**
Shared look rate (count/min)	Mean (SD)	0.21 (0.42)	0.25 (0.34)
	Median (IQR)	0.00 (0.00, 0.21)	0.00 (0.00, 0.48)
	[Range]	[0.00, 2.32]	[0.00, 1.35]
Shared attention rate (count/min)	Mean (SD)	3.1 (1.3)	3.0 (1.6)
	Median (IQR)	3.1 (2.2, 3.7)	2.9 (1.9, 4.1)
	[Range]	[0.0, 7.7]	[0.0, 6.7]
Shared attention intensity (% time)	Mean (SD)	67 (16)	66 (21)
	Median (IQR)	69 (60, 79)	69 (59, 80)
	[Range]	[0, 95]	[0, 94]

Of the potential confounders, maternal depression, an infant having a father with a “manual” occupation and maternal age were associated with measures of JA. Depression scores tended to be negatively associated, or nearly so, with shared look rate (*p* = 0.332 at 32–40 weeks gestation; *p* = 0.052 at eight months postnatal) and shared attention rate (*p* = 0.033 at 32–40 weeks gestation; *p* = 0.093 at 8 months postnatal), but positively associated with shared attention intensity (*p* = 0.002 at 32–40 weeks gestation; *p* = 0.030 at eight months postnatal). EPDS depression scores are summarised and compared between the case–control groups in Table 3.

**Table 3:** Mean (SD) Edinburgh Postnatal Depression Scale (EPDS) scores at 32–40 weeks gestation and 8 months postnatal among controls and cases

**Time EPDS score recorded**	**Control (n = 106)**	**Case (n = 53)**	**p-value**^**a**^
32-40 weeks gestation^b^	5.9 (4.0)	8.7 (6.1)	0.004
8 months postnatal	5.0 (4.6)	6.9 (5.7)	0.038
Mean of 32–40 weeks gestation and 8 months postnatal	5.4 (3.7)	7.8 (5.3)	0.005

^a^p-values calculated using t-tests.

^b^EPDS scores at 32–40 weeks gestation were missing for 6 controls and 2 cases.

Because these two depression scores were strongly positively correlated with each other (Spearman’s ρ = 0.59), and neither showed a consistently stronger association than the other with the shared attention measures, we combined these into a single mean depression score, which was consistently significantly associated with all three joint attention measures (all *p* < 0.05). All subsequent models were adjusted for this mean depression score.

In addition, having a father with a “manual” occupation was negatively associated with shared attention rate (*p* = 0.073), maternal age was negatively associated with shared attention intensity (*p* = 0.078), and log interaction duration was positively associated with shared attention intensity (*p* = 0.075). Models predicting shared attention rate were therefore additionally adjusted for paternal occupation, while models predicting shared attention intensity were additionally adjusted for maternal age and log video duration, respectively. However, adjusting for these potential confounders had no substantial effect on the results presented in Table 4.

**Table 4:** Logistic regression analyses showing odds ratios (OR) with 95% confidence intervals (CI) and p-values relative to controls (n = 106) between joint attention measures and case–control status, including overall case status and diagnostic subgroups

		**N**	**OR (95% CI) per SD**		
			**p-value**		
			**Shared look rate**	**Shared attention rate**	**Shared attention intensity**
All cases		53	1.2 (0.9, 1.7)	1.0 (0.7, 1.5)	0.8 (0.6, 1.2)
			p = 0.269	p = 0.892	p = 0.329
Diagnostic subgroup					
	Disruptive behaviour disorders (any ADHD + any oppositional-conduct disorder)	32	1.3 (0.9, 1.9) p = 0.156	1.0 (0.7, 1.6) p = 0.900	0.7 (0.5, 1.1) p = 0.132
	Any ADHD disorder	15	1.1 (0.6, 1.7) p = 0.719	0.9 (0.5, 1.6) p = 0.820	0.6 (0.4, 1.1) p = 0.095
	Any oppositional-conduct disorder	24	1.5 (1.0, 2.3) p = 0.041	1.1 (0.7, 1.8) p = 0.699	0.8 (0.5, 1.2) p = 0.259
	Pervasive development disorder	5	1.2 (0.4, 2.0) p = 0.655	0.6 (0.2, 1.6) p = 0.328	1.6 (0.5, 6.9) p = 0.438
	Any emotional disorder	24	1.3 (0.8, 1.9) p = 0.217	1.2 (0.7, 2.2) p = 0.495	0.9 (0.5, 1.7) p = 0.761

All models were adjusted for sex and potential confounders (see main text for details). To aid interpretation, odds ratios have been scaled to represent the increase in the odds of being a case associated with a 1 SD increase in each joint attention measure.

None of the three joint attention measures showed a significant association with the primary outcome of case–control status (Table 4). Only shared look rate predicted any of the exploratory sub-diagnosis outcomes, being positively associated with diagnosis of any oppositional-conduct disorder (*p* = 0.041). A positive difference of one SD in the shared look rate (equivalent to an additional two shared looks every five minutes; Table 2) predicted an approximately 50% increase (OR [95% CI]: 1.5 [1.0, 2.3]) in the odds of diagnosis with any oppositional-conduct disorder (Table 4). An alternative way of viewing this association is that subjects diagnosed with any oppositional-conduct disorder shared looks with their caregiver more frequently, on average, than did controls (mean looks/min in cases: 0.35; controls: 0.21).

## Discussion

Based on a large cohort of infants, we investigated whether it was possible to predict diagnosis of psychiatric disorders from analysis of mother-infant joint attention behaviours in social-communicative interaction at 12 months.

Specifically, we examined three JA behaviours: shared look rate (count/min), shared attention rate (count/min)

and shared attention intensity (% time). There was no evidence that JA at one year strongly predicts psychopathology at age seven. None of the three JA measures showed a significant association with the primary outcome of case–control status. Only shared look rate predicted any of the exploratory sub-diagnosis outcomes and was found to be positively associated with later oppositional-conduct disorders.

It is possible that other associations exist but were not detected in this study. There are a variety of explanations for this. Firstly, the methodological shortcomings of our study need to be considered. Our study was powered to detect only strong effects which was something that we could not modify in this exploratory study. With regards to the sub-diagnoses, some of these had very small sample sizes, so associations would have had to be very strong indeed to have been detected. Potential existence of weaker associations, not detectable by the present exploratory study, may be due to the case group being too broadly defined, so that true associations between JA measures and sub-diagnoses might be hidden due to being combined in the case group with diagnoses with no association or opposite associations. However, this explanation is not well supported by the results of the tests for association between the three joint attention measures and the five sub-diagnoses, since only one of the 15 tests – between the rate of shared looks and any oppositional-conduct disorder – was significant, which is close to the number expected due to chance alone.

The angle of the camera recording the videos is a potential limitation as discussed earlier in the methods section. Additionally, it is possible that the structure of the book situation (caregiver asked to share a picture book with their infant and engage their child in this activity as they would at home) reduced the social demand of the context and modulated the child’s activity and behaviour masking possible associations between JA behaviours and later diagnosis. Therefore, the scaffolding measures adopted during the task by the caregiver is creating a more controlled and limiting environment than say a free play situation. Nevertheless, the observers were able to assess variation in JA levels between videos, and do this with high inter-rater reliability. However, it may simply be the case that there really are no other associations or significant predictors of later diagnoses observable from the JA behaviours and the single significant result was due to chance. There is also the issue of accuracy of the DAWBA version which was used in identifying psychopathology in the present study. It has been considered limited in its ability to identify autism – the five cases of PDD were not identified as having an ASD by the clinicians. The PDD diagnoses were not made using the specific section of the DAWBA which was developed later, instead the diagnoses were made incidentally from other questions. In sum, we found no evidence that JA at one year strongly predicts psychopathology as a whole at age seven.

From previous literature, it is possible that the reason no association was found between JA and psychopathology in the present early sample is that psychiatric disorders, including ADHD, have been found to take different trajectories from infancy to adolescence [50,51]. Shared attention also failed to predict autism (one of the five types of pervasive developmental disorders) which may be due to the caregiver compensating for the infant’s behaviour [52]. Previous studies have found shared attention or JA difficulties in infancy [30] and it is even argued to be one of the earliest signs of autism [31,32]. The small sample size of only five cases may be a reason for the lack of significant findings with respect to the prediction of later diagnosis of pervasive development disorder. It is also possible that joint attention between caregivers and infants can neither explain nor predict later psychopathology, so that the association between shared look rate and any oppositional-conduct disorder was a chance result. Additionally, there may be cases within the control group and vice versa [52,53], since both under-diagnosis and over-diagnosis routinely occur in ADHD [54,55]. Surprisingly our study found that, of the three joint attention behaviours, only shared look was positively associated with later oppositional-conduct disorders and none of the joint attention behaviours predicted or were associated with ADHD. A potential interpretation of this finding is that the mothers of the infants later diagnosed with ADHD were perhaps (even on an unconscious level) controlling the infants impulsivity and “watching” the infants behaviour. It may also be an indication of extroversion in both the mother and infant or infant alone. Previous research however has shown in *older* children aged between 4–8 years that, compared with controls, children with oppositional defiant disorder expressed lower levels of affection back towards their mothers; those with high levels of callous-unemotional traits showed significantly lower levels of affection than the children lacking these traits. The former group exhibited lower levels of eye contact toward their mothers. These impairments were found to be independent of maternal behaviour. No group difference in affection and eye contact expressed by the mothers was found [49].

We have presented a case–control study nested within a prospective longitudinal cohort study. The longitudinal nature of the study is one of its main strengths. Previous studies have either been retrospective or have sampled high risk referred children or siblings of affected individuals. Retrospective studies are limited in that they are primarily based on parental reports which are often biased and subject to recall/memory problems. Here we report the first study of the utility of measures of joint attention in early mother-infant interaction in predicting later onset of childhood psychopathology, based on a large cohort of infants from the ALSPAC community-based cohort. Another important strength of the present study is that all the children in the study received an independent psychiatric assessment at age seven years using the DAWBA [44]. Lastly, we made a partial adjustment for caregiver psychopathology via the maternal depression rating which is important to strengthen the conclusions we draw from our findings given that there is much evidence strongly indicating the impact of maternal psychopathology on infant cognitive and psychological development [56,57] and behaviour [58]. On the other hand, one study found that maternal depression (whether prior to the birth, postpartum, or at nine months) had little impact on JA between a caregiver and nine month old infants and therefore, relationships between JA and maternal behaviour reflect infants’ social interactions with their mothers, not depression *per se*[59,60].

A future study could also improve the quality of video recording to ensure that parent and child faces are always in optimum view. A new larger cohort, comprising ‘at-risk’ infants using more task conditions (i.e. play, feed, etc.), could be implemented using a placement of video equipment which would enable the capturing of more information. These more naturalistic settings might reveal more than the constrained setting of TIM and a previous study has found it to be an effective method [61]. It remains to be established whether analyses of this kind can contribute to the development of screening instruments for disorders amenable to early intervention [61].

## Conclusions

In conclusion, in this study JA behaviour did not predict later psychopathology. There was, however, a positive association between the rate of shared looks and later development of any oppositional-conduct disorder. In this opportunistic study, where neither the sample size nor the set-up was ideal, no strong associations were found between JA and psychopathology. However weaker associations are still plausible, and might be detectable via a larger and more tailored study. There is scope for improving our knowledge of the mechanisms underlying the various disorders as well as informing the development of effective treatments and improving the reliability of screening instruments which may be of potential value for disruptive behaviour disorders amenable to early intervention [62].

## Competing interests

The authors declare no conflicts of interests.

## Authors’ contributions

CSA drafted the manuscript with PJ. PJ performed the statistical analysis under AM's supervision. CP, EL and EK supervised the observations. AM; HM; JG; CP and CG designed the study. PW designed study and is the principal investigator and guarantor for the contents of this article. All ten authors reviewed the manuscript.

## Financial competing interests

ALSPAC currently receives core support from Wellcome Trust, Medical Research Council and the University of Bristol. This project was specifically funded by small grants from the Yorkhill Children’s Foundation, the Gillberg Neuropsychiatry Centre and the Waterloo Foundation.

## Non-financial competing interests

The authors declare no non-financial competing interests.

## Pre-publication history

The pre-publication history for this paper can be accessed here:

http://www.biomedcentral.com/1471-2431/13/147/prepub

## Supplementary Material

Additional file 1 Definitions of each of the elements of joint attention behaviours. Click here for file
