# Supplementary material for: Prediction of 7-year psychopathology from mother-infant joint attention behaviours: a nested case–control study
Source: BMC Pediatr. 2013 Sep 24;13:147. doi: 10.1186/1471-2431-13-147 (PMC3848970; doi:10.1186/1471-2431-13-147)
Supplement: Additional file 1 — Definitions of each of the elements of joint attention behaviours. [file 1471-2431-13-147-S1.doc]

Additional file 1

*1. Joint Attention*

Definition: joint attention can be understood to be the state of shared awareness of self and others attention, purposes, feelings and meanings in relation to a shared focus (to an object, action, event, experience or person in the world). We can only judge this through observable behaviors, in particular, through sequences of observable behaviors.

Joint attention can be judged by observing a sequence of events in which:

1. The child and parent look at each other (initiation phase)

2. Followed by both looking at or acting on, or acting in relation to, a joint object or event (shared attention/action/communicative act phase)

3. Followed by the child and parent looking back at each other (confirm/check joint response phase)

Each event will be recorded, even if the sequence is not complete, for example, a 1 and 2 may be recorded without a 3 occurring.

*2. Parent Use of Child’s Name*

Definition: Instances of parent uttering child’s name.

*3. Parent Affective Communicative Function*

Definition: where parent seems to intend to effect an emotional state or feeling in the other participant [e.g. effecting that the other person feels relaxed, relieved, happy, reassured].

*4. Parent Informative Communicative Function*

Definition: Where the parent seems to intend to inform or teach the infant about something. This could be labeling ‘that’s a dog’, or a comment ‘you’ve got one of these at home’.

*5. Parent Directive and Attention Seeking Communicative Function*

Definition: Where the parent seems to intend to gain, maintain or direct the child’s attention, or direct the child’s actions – e.g. ‘look, what’s this?’, or ‘show me your shoe’, or ‘don’t tear it’.

Can be separated into:

Attention seeking - Where parent seems to intend to gain, maintain or direct the child’s attention, e.g. ‘oh, what’s this’.

Parent directive - Where the parent seems to intend direct the child’s actions and purposes (parent has an expectation that child will understand) – e.g. ‘show me your shoe’, or ‘don’t tear it’.

*6. Infant Directive Communicative Function*

Definition: Where the infant seems to intend to gain, maintain or direct the adult’s attention, or direct the adult’s actions – such as reaching out to an object and then looking at the adult and back to the object.

*7. Infant Contingent Responsiveness to Adult*

Definition: Where the infant’s expressiveness or action seems to be contingently linked to a communicative move or action of the adult – such as where the adult points to something in the book (oh, what’s this?) and the infant looks to where the adult is pointing.

*8. Infant Compliance to Adult Directive*

Definition: Where the infant seems to respond appropriately in accordance with a directive communicative purpose of the adult – such as where adult says ‘show me your shoe’ and the infant contingently points to their shoe, and probably then looks at the adult.

*9. Adult Contingent Responsiveness to Infant*

Definition: Where the adult’s expressiveness or action seems to be contingently linked to a communicative move or action of the infant – such as where the infant points to something in the book and the adult looks to where the infant is pointing.

*10. Adult Compliance to Infant directive*

Definition: Where the adult seems to respond appropriately in accordance with a directive communicative purpose of the infant – such as where infant reaches out to something as if they want to go to it, and the adult complies by reaching out and lifting the infant towards the object.

*11. Infant Gaze at Book*

Definition: the infant gazes at the book, including a code for when the view of the infant is blocked.

*12. Infant Following Adult Focus*

Definition: The infant looks at the adult and then looks to object of adult’s focus.

*13. Infant Protodeclarative Pointing*

Definition: The infant points at an object for the purpose of making the parent look at the object.

*14. Infant Pointing*

Definition: The infant points at an object without any intention of making others attend to object.

*15. Infant Smile*

Definition: Code when the infant smiles.

*16. Infant Non-Response to Name*

Definition: The infant’s name is said by the parent, but the infant does not make an apparent response.

*17. Infant Imitation*

Definition: The infant imitates the parent’s actions. This can be coded as verbal or physical.

*18. Infant Sensory Sensitivities*

Definition: Instances of the infant appearing overly sensitive to the environment. For example, startle reactions, shunning being touched, or quick adverse facial expressions to visual and audio stimuli.

*19. Response to Bell*

A loud bell used in another part of the 1-year Children in Focus assessment is often audible in the ALSPAC videos.

Definition: Does infant respond either physically or verbally to the bell? Make note if bell is not rung.

*20. Infant’s Co-ordinated Fine Motor Function (page turning)*

Definition: When turning pages, does the infant use a pincer (finger and thumb), tripod (fingers clasped together) or open hand.

*21. Regulated Behavior*

Smooth, coordinated behavior that is directed to achieve a goal, be that engaging in the book task (via communicative exchanges with the parent or by manipulating the book), communicating or playing with the parent, or communicating with the assessor.

*22. Dysregulated Behavior*

Behaviors that do not serve any task-related or communicative (in the sense of positive communication) function. The infant’s behavior becomes less fluent and more fragmented. The level of activity may either begin to increase so that he appears agitated or becomes overtly distressed and fussy. Alternatively he may lose physical tone and flop down on the sofa or the parent.

Behaviors scored under this dimension include: rapid head turns (the infant rapidly turns his head in either direction. The action is too rapid for the infant to visually fixate on any object), agitated body movements or moving quickly around the sofa or onto the floor, arching the body or straining the neck or twisting away from the parent/task (the infant tries to distance himself by turning and twisting away from the mother. The infant’s head is averted sideways from the adult, with gaze directed away from the adult. The infant may actively push away from the parent), losing tone (relative to that held previously. The infant’s body appears to lose tone and he sits inert and motionless for a time, or flops himself down and remains relatively motionless.).

*23. Regulating Behavior*

Parent: The infant actively seeks contact with the parent and cuddles in close to him/her. He may push himself closer to her if already on the parent’s lap, or he may turn to face the parent and touch his/her face/head.

Self:

• Touches head/face- the infant touches or rubs his face or head (e.g. rubs eyes or ears, pulls own hair).

• Touches Body - The infant actively touches or rubs some part of his clothing or body (e.g. his shirt or his hair or his ears). To be sure that this is self-touching observe any opening and closing of the fingers as the infant is fingering or stroking his clothes. Self clasp - the infant clasps his hands together or wraps his arms around himself as in a self hug.

• Oral Self - The infant brings to his mouth or sucks his own body e.g. a thumb, finger, or fist. There must be skin and hand to mouth contact.

Object:

• Touches Object - the infant seeks comfort through touch of a toy. This may co-occur with ‘oral self’ or ‘oral object’ (e.g. sucking thumb while holding a toy to the face).

• Oral Object - The infant brings to his mouth or sucks something other than his own body e.g. a toy, a dummy or clothing.
